# Chromatic Pupillometry Findings in Alzheimer’s Disease

**DOI:** 10.3389/fnins.2020.00780

**Published:** 2020-08-11

**Authors:** Martina Romagnoli, Michelangelo Stanzani Maserati, Maddalena De Matteis, Sabina Capellari, Michele Carbonelli, Giulia Amore, Gaetano Cantalupo, Corrado Zenesini, Rocco Liguori, Alfredo A. Sadun, Valerio Carelli, Jason C. Park, Chiara La Morgia

**Affiliations:** ^1^IRCCS Istituto Delle Scienze Neurologiche di Bologna, UOC Clinica Neurologica, Ospedale Bellaria, Bologna, Italy; ^2^Department of Biomedical and Neuromotor Sciences, University of Bologna, Bologna, Italy; ^3^Division of Child Neuropsychiatry, Department of Surgical Sciences, Dentistry, Gynaecology and Paediatrics, University of Verona, Verona, Italy; ^4^Department of Ophthalmology, Doheny Eye Institute, University of California, Los Angeles, Los Angeles, CA, United States; ^5^Department of Ophthalmology and Visual Sciences, University of Illinois at Chicago, Chicago, IL, United States

**Keywords:** chromatic pupillometry, Alzheimer’s disease, melanopsin retinal ganglion cells, pupillary light reflex, post-illumination pupil response, pupil

## Abstract

Intrinsically photosensitive melanopsin retinal ganglion cells (mRGCs) are crucial for non-image forming functions of the eye, including the photoentrainment of circadian rhythms and the regulation of the pupillary light reflex (PLR). Chromatic pupillometry, using light stimuli at different wavelengths, makes possible the isolation of the contribution of rods, cones, and mRGCs to the PLR. In particular, post-illumination pupil response (PIPR) is the most reliable pupil metric of mRGC function. We have previously described, in post-mortem investigations of AD retinas, a loss of mRGCs, and in the remaining mRGCs, we demonstrated extensive morphological abnormalities. We noted dendrite varicosities, patchy distribution of melanopsin, and reduced dendrite arborization. In this study, we evaluated, with chromatic pupillometry, the PLR in a cohort of mild-moderate AD patients compared to controls. AD and controls also underwent an extensive ophthalmological evaluation. In our AD cohort, PIPR did not significantly differ from controls, even though we observed a higher variability in the AD group and 5/26 showed PIPR values outside the 2 SD from the control mean values. Moreover, we found a significant difference between AD and controls in terms of rod-mediated transient PLR amplitude. These results suggest that in the early stage of AD there are PLR abnormalities that may reflect a pathology affecting mRGC dendrites before involving the mRGC cell body. Further studies, including AD cases with more severe and longer disease duration, are needed to further explore this hypothesis.

## Introduction

Melanopsin retinal ganglion cells (mRGCs) are intrinsically photosensitive RGCs because of the expression of the photopigment melanopsin (Berson et al., 2002; Hannibal et al., 2002; Hattar et al., 2002). These cells contribute to non-image forming functions of the eye including circadian photoentrainment [projecting via the retino-hypothalamic tract (RHT) to the suprachiasmatic nucleus (SCN) of the hypothalamus] and regulation of the pupillary light reflex (PLR) [via projections to the olivary pretectal nucleus (OPN)] (Sadun et al., 1984; Hannibal et al., 2004, 2014; Baver et al., 2008; Chen et al., 2011; Li and Schmidt, 2018).

Neurodegenerative disorders, including Alzheimer’s disease (AD), are characterized by prominent circadian and sleep dysfunction even in the early phase of the disease (Uddin et al., 2020). Melanopsin retinal ganglion cell loss demonstrated in post-mortem AD retinas may contribute to the circadian and sleep problems documented in these patients (La Morgia et al., 2016, 2017).

Amyloid plaques have been detected in AD retinas (Koronyo et al., 2017) and amyloid pathology can also affect mRGCs, suggesting a specific mechanism of neurodegeneration independent from the aging process (La Morgia et al., 2016). Moreover, extensive morphological abnormalities with dendrite varicosities, patchy distribution of melanopsin, and reduced dendrite arborization were noted in remaining mRGCs of AD retinas (La Morgia et al., 2016).

The function of mRGCs is, however, difficult to explore *in vivo*, since these cells represent a small subgroup (about 1%) of the regular RGCs, and also mRGCs receive some input from rods and cones (Hannibal et al., 2017). Chromatic pupillometry protocols have been developed to isolate the contribution of mRGCs to the PLR and to assess *in vivo* the function of mRGCs (Kardon et al., 2011; Park et al., 2011; La Morgia et al., 2018). These protocols are based on light stimuli at different wavelengths and with light adaptation conditions aimed at isolating the contribution of single photoreceptors, taking into account that mRGCs are maximally sensitive to blue light at 480 nm (Berson et al., 2002). It has been shown that the post-illumination pupil response (PIPR) is the most reliable pupil metric of mRGC function (Adhikari et al., 2015b).

Previous studies investigated the presence of pupil abnormalities in AD patients but they used different visual stimuli, heterogeneous protocols, and results were not consistent (Chougule et al., 2019). Pre-symptomatic cases (Oh et al., 2019; Van Stavern et al., 2019) and, recently, early AD cases (Kawasaki et al., 2020) were evaluated with chromatic pupillometry to isolate the mRGC contribution.

The present study was designed to evaluate the PLR, and in particular the mRGC-mediated contribution, in AD. We here report chromatic pupillometry findings using a previously published protocol (Park et al., 2017) in a cohort of 26 mild-moderate AD patients and 26 controls for which a detailed neuro-ophthalmological evaluation has been performed.

## Methods

### Study Participants

This is a cross-sectional study and follows the STROBE guidelines (von Elm et al., 2007). We included AD patients and healthy controls, evaluated between June 2017 and February 2020 at the IRCCS Institute of Neurological Sciences of Bologna. All subjects gave written informed consent for the prospective collection of clinical data, data analyses, and publication. The study was conducted in agreement with the Declaration of Helsinki and approved by the local ethical committee (EC Interaziendale Bologna-Imola #16032) and within the framework of the research project supported by the Italian Ministry of Health, GR-2013-02358026 to CLM. We included patients with a diagnosis of AD according to Dubois criteria (Dubois et al., 2014) and National Institute of Neurological and Communication Disorders–Alzheimer’s Disease and Related Disorders Association criteria (NINCDS-ADRDA) (McKhann et al., 1984) at mild–moderate stage [Mini-Mental State Examination (MMSE) score between 11 and 26] (Folstein et al., 1975).

The absence of cognitive dysfunction was ascertained in the control group.

Exclusion criteria for both control and AD groups were: spherical or cylindrical refractive errors more than 3 or 2 diopters, respectively; presence of posterior pole pathology including age-related macular degeneration and known optic neuropathies (including open-angle glaucoma); ocular pressure more than 20 mmHg; severe lens opacity and/or retinal detachment and/or vascular retinal pathology (including diabetic retinopathy); history of ophthalmologic surgery, except for uncomplicated cataract surgery, performed at least 6 months previously; shift-workers in the last year; travels through more than one time zone during the last 3 months.

All study participants completed self-administered questionnaires including Epworth Sleepiness Scale (ESS), Pittsburgh Sleep Quality Index (PSQI), Berlin questionnaire, and Beck Anxiety (BAI) and Depression Inventory (BDI) (Beck et al., 1961, 1988) to evaluate the possible occurrence of sleep disturbances. For the control group, exclusion criteria included also the presence of the following abnormal scores at sleep and mood questionnaires: excessive daytime sleepiness as assessed by the ESS (Vignatelli et al., 2003); presence of sleep disturbances as determined by the PSQI (Buysse et al., 1989); abnormal scores on the BAI (Beck et al., 1988) and BDI (Beck et al., 1961) tests.

All subjects underwent an extensive neuro-ophthalmological evaluation including visual acuity testing, tonometry, fundus examination, Ishihara color vision test, and OCT examination. OCT examination was performed using SS (Swept-Source)-OCT with the deep range imaging (DRI) Triton OCT (Topcon, Japan) using the 3DWide 12 × 9 mm scan protocol including segmentation analysis. We evaluated the average and 4 individual quadrants (temporal, superior, nasal, and inferior) peripapillary Retinal Nerve Fiber Layer (pRNFL) thickness, and the average and 6 individual macular sectors (superotemporal, superior, superonasal, inferonasal, inferior, and inferotemporal) Ganglion Cell-Inner Plexiform Layer thickness (GCL + defined as the thickness from the inner boundary of the GCL to the outer boundary of the inner plexiform layer [IPL]). OCT scans were acquired by the same experienced operator (MC) and poor-quality images (quality index less than 60), segmentation or centered errors, presence of any optic disk abnormalities potentially interfering with the goodness of OCT examination (presence of dysmorphic or tilted optic disk) were rejected from OCT data analysis. Moreover, AD patients performed neuropsychological evaluation and the MMSE corrected (MMSEc score) was obtained for all of them. We also collected all the clinical information available, including concomitant medications potentially impacting on pupil function (Chougule et al., 2019; Kelbsch et al., 2019), for both controls and AD patients.

### Chromatic Pupillometric Protocol

#### Apparatus, Stimuli, and Procedures

A Ganzfeld ColorDome full-field stimulator (Espion V6, ColorDome Desktop Ganzfeld; Diagnosys LLC, Lowell, MA, United States) was used for the chromatic pupillometry test. Participants were dark-adapted for 10 min prior to start of the test. With the exclusion of patients for which only one eye was eligible for the study, we tested the dominant eye, and the contralateral eye was patched for monocular testing (65% of the tested eyes were right eyes). Colored light stimuli were presented to the tested eye and the pupil responses were recorded from the same eye using the Ganzfeld system equipped with an integrated pupillometer. The complete pupillometric protocol for isolating the rod-, mRGC-, and cone-contribution is described in details elsewhere (Park et al., 2011). For this study we considered the following conditions, as previously reported (Park et al., 2017):

1. Rod-condition: low luminance (0.001 cd/m^2^) blue flash presented in the dark;
2. Melanopsin-condition: photopically-matched red and blue stimuli (450 cd/m^2^) presented in the dark;
3. Cone-condition: red flash (10 cd/m^2^) presented against the rod-suppressing blue adapting field (6 cd/m^2^).

Stimuli consisted of short wavelength (blue, dominant wavelength of 460–485 nm; mid = 472 nm) and long wavelength (red, dominant wavelength of 620–645 nm; mid = 632 nm) light-flashes of 1 s duration. The integrated pupillometer system measured the pupil diameter at a 100 Hz sampling frequency. The interstimulus interval (ISI) was 20 s for the rod- and cone-conditions (for both red and blue stimuli), while for the melanopsin-condition ISI was 30 s for red stimulus and 70 s for the blue one. All recordings were completed in the same order with the red stimulus followed by the blue. For all three conditions, each stimulus was presented three times consecutively and the individual responses were obtained by their average recording. Participants were instructed to keep their eyes open during the duration of the light stimuli as well as following the stimuli. Participants who blinked frequently during the recordings were given another opportunity to repeat the measurements. Pupil traces with excessive artifacts due to long eye blinks or eye closure were excluded from subsequent pupillometric data analysis.

#### Data Analysis

Data were analyzed using custom scripts programmed in MATLAB (MathWorks Inc., Natick, MA, United States), which allowed for semi-automated analysis. PLR was normalized by the median steady-state (baseline) pupil size during the 2 s preceding each stimulus onset in order to minimize the effects of inter-subject differences in the baseline pupil size.

The following pupillometric parameters were calculated:

I. Transient PLR amplitude (or Transient Peak Amplitude) was defined as the difference between the normalized baseline and the minimum normalized PLR after stimulus onset (pupil maximum constriction);
II. For the melanopsin-condition, the PIPR was used for evaluating the mRGC sustained response. PIPR parameter was defined as the difference between the normalized baseline and the median normalized PLR measured over a 5 to 7 s time interval from stimulus offset. In particular, we evaluated PIPR from the blue and the red photopically-matched stimuli, and also the difference between the blue PIPR and the red one (PIPR_Normalized_ = PIPR_Blue_ - PIPR_Red_).

### Statistical Analysis

The Shapiro-Wilk and Kolmogorov–Smirnov tests were performed to assess the normal distribution and graphic inspection of the data. Chi-square and independent-*t* tests were used to compare variables among groups. For continuous variables (pupillometric parameters), z-scores (standard scores) were also calculated. Levene’s test was used to assess the equality of variances for mRGC sustained response for control and AD groups. Comparisons between groups for all pupillometric variables, measured under rod-, melanopsin-, and cone-mediated conditions, were computed by means of analysis of covariance (ANCOVA) with age as the covariate. Moreover, the *p*-value for interaction age × group was computed from the log-likelihood ratio test comparing ANCOVA models with and without the interaction term, and stratified β coefficients (95% Confidence Interval, 95% CI) for the variables turning out to be effect modifiers (*p*-value for interaction < 0.15) were presented.

For OCT data, we followed “one-eye” approach by evaluating the eye tested by chromatic pupillometry. Pearson correlation coefficients were used to measure the degree of association between pupillometric parameters and clinical data (OCT measures, MMSEc score, and disease duration) in control and Alzheimer’s groups. Statistical analyses were performed using SPSS (SPSS Inc., IBM, Chicago, IL, United States) and Stata SE (StataCorp, College Station, TX, United States) softwares.

## Results

This study included 26 mild-moderate AD patients from 52 to 88 years of age (69.3 ± 7 years) and 26 healthy participants (controls) from 58 to 82 years of age (70.2 ± 11 years). The demographic and clinical data of the two groups are shown in Table 1. Controls and AD patients did not significantly differ in terms of age and gender (gender, *p* = 0.58; age, *p* = 0.75).

**TABLE 1:** Sociodemographic data.

	**Controls**	**Alzheimer’s**	***p*-value**
***N***	26 (50%)	26 (50%)	
**Gender**			
Male	11 (42.3%)	14 (53.8%)	0.58
Female	15 (57.7%)	12 (46.2%)	
**Age | Age-class**	69.3 ± 7	70.2 ± 11	0.75 | 0.16
50–59 years	3 (11.5%)	6 (23.1%)	
60–69 years	10 (38.5%)	4 (15.4%)	
70–79 years	10 (38.5%)	9 (34.6%)	
80–89 years	3 (11.5%)	7 (26.9%)	
**MMSEc**	/	20.7 ± 4	/
		(17.5–24.8)	
**Disease duration**	/	3.8 ± 2.9	/
		(2–4.2)	

*Values are given as n (%) or mean ± standard deviation (interquartile range, Q1–Q3). MMSEc, Mini Mental State Examination corrected score. Chi-square test was performed with categorical variables and independent-t test was performed with continuous variables.*

Raw pupil traces from two controls showed excessive blink artifacts under the rod- (blue flash) and melanopsin- (blue flash) conditions and were removed from data analysis. Further, four control and six AD pupil traces under the cone-condition showed too many artifacts, were not reliable, and thus were removed from data analysis.

**FIGURE 1:**
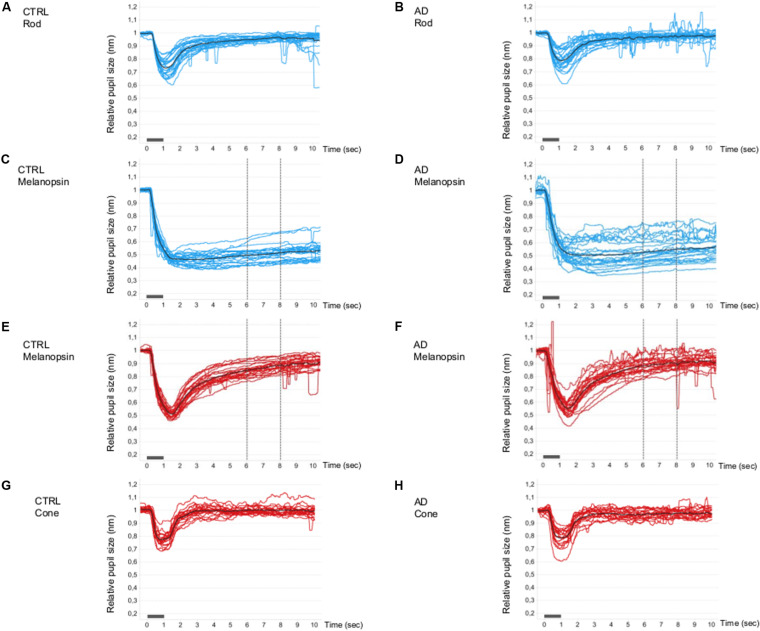
Single and mean pupillometric waveforms for the rod, melanopsin, and cone conditions in controls and AD. Pupillometric traces obtained under the rod **(A,B)**, melanopsin (**C,D**, blue and **E**,**F**, red), and cone **(G,H)** conditions of the chromatic pupillometric protocol. Light blue **(A–D)** and red **(E–H)** traces represent single individuals, while black traces **(A–H)** represent the mean waveforms for each group (**A,C,E,G** for the control group; **B,D,F,H** for Alzheimer’s group). The vertical dotted lines indicate the time interval (5–7 s from stimulus offset) in which the melanopsin-mediated (sustained) amplitude (PIPR, Post-Illumination Pupil Response, 450 cd/m^2^) was measured. The light stimulus onset and offset are represented by the gray boxes along the x-axes.

The single normalized pupil traces (PLR curves) under all conditions are shown in Figure 1. For the rod- (Figures 1A,B) and cone- (Figures 1G,H) conditions, the PLR is characterized by a rapid transient constriction followed by a relatively rapid return to the baseline both in controls (Figures 1A,G) and in AD (Figures 1B,H).

Under the melanopsin-condition (blue flash), in both controls (Figure 1C) and AD (Figure 1D), the PLR is characterized by an initial transient constriction followed by a sustained constriction (PIPR) during the 5–7 s time interval from light-stimulus offset. Melanopsin-mediated sustained response is more variable in the AD group (Figure 1D) compared to the control group (Figure 1C). In fact, the estimated population variances of the PIPR at 5-s from stimulus offset of the two groups were statistically different (Levene’s test: SD control = 0.03, SD AD = 0.06; *p* = 0.018) and, in particular, the variability in the AD group resulted significantly greater. Moreover, five AD patients showed a PIPR lower more than 2 SD from the control mean value. Under the melanopsin-condition (red flash), in both controls (Figure 1E) and AD (Figure 1F), the elicited PLR is characterized by an initial transient constriction, followed by a smaller sustained response with a reduced amplitude in the AD group compared to controls.

**FIGURE 2:**
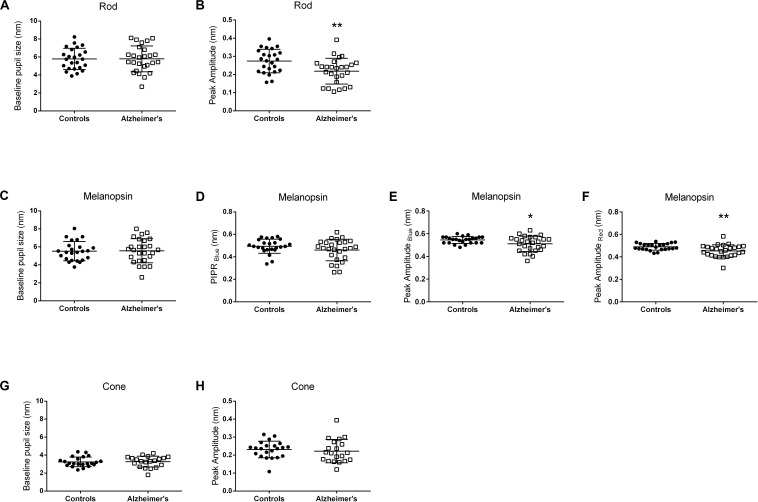
Pupillometric parameters for controls and AD. Panels show scatterplots with horizontal solid line represents the mean and error bars representing standard deviations for each group. **(A,B)** Show the results for the Rod-condition; **(C–F)** Show the results for the Melanopsin-condition [**(C–E)** 450 cd/m^2^, 472 nm-blue; **(F)** 450 cd/m^2^, 632 nm-red]; **(G,H)** Show the results for the Cone-condition. **(A,C,G)** Show normalized pupil size at baseline. **(B,E,F,H)** Show normalized transient peak amplitude. **(D)** Show normalized melanopsin-mediated Post-Illumination Pupil Response (PIPR_Blue_). Peak amplitude (transient peak amplitude) was defined as the difference between the normalized baseline pupil size and the median normalized PLR at the point of maximum pupillary constriction after stimulus onset. PIPR was defined as the difference between the normalized baseline pupil size and the median normalized PLR measured over a 5 to 7 s time interval from stimulus offset. Significant different between controls and AD patients are indicated by an asterix symbol above the groups. *p < 0.05; **p < 0.01.

The individual pupillometric parameters for controls and AD for the three conditions are provided in Figure 2. There was no difference between AD and control groups in terms of baseline normalized pupil size under any of the three conditions (Figures 2A,C,G and Supplementary Table 1). PLR transient amplitude (Figures 2B,E,F and Supplementary Table 1) was significantly decreased under rod- (*p* = 0.006) and melanopsin- (blue flash, *p* = 0.02; red flash, *p* = 0.006) conditions in AD compared to controls. PIPR_Blue_ in the melanopsin-condition was not significantly different between AD and controls (Figure 2D and Supplementary Table 1). PLR transient amplitude was not significantly different between AD and controls under the cone-condition (Figure 2H and Supplementary Table 1).

**FIGURE 3:**
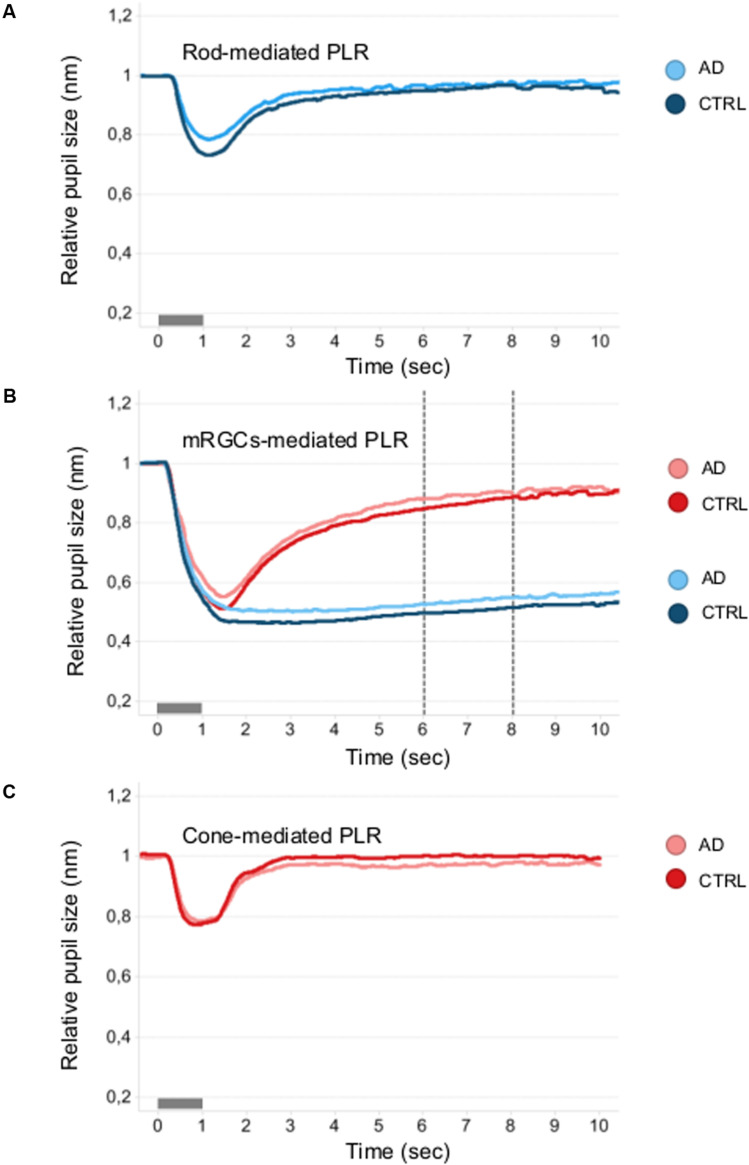
Mean pupillometric waveforms obtained under the rod, melanopsin, and cone conditions in controls and AD. **(A)** Shows PLR measured under the rod-condition with the short- (blue) wavelength flashes (0.001 cd/m^2^) presented in the dark, for comparison between control (blue mean trace) and AD (azure mean traces) groups. **(B)** Shows PLR measured under the melanopsin-condition, including the two photopically-matched intense long- (red) and short- (blue) wavelength flashes (450 cd/m^2^) presented in the dark, for comparison between control (red and blue mean traces) and AD (pink and azure mean traces) groups. The vertical dotted lines indicate the time interval (5–7 s from stimulus offset) over which the melanopsin-mediated (sustained) amplitude (PIPR) was measured. **(C)** Shows PLR measured with the long- (red) wavelength flashes (10 cd/m^2^) presented against the rod-suppressing blue adapting field (6 cd/m^2^) for comparison between control (red mean trace) and AD (pink mean trace) groups.

Figure 3 shows the mean normalized pupil traces of rod- (Figure 3A), melanopsin- (Figure 3B), and cone- (Figure 3C) conditions for each subject group. We failed to observe any difference in terms of PIPR_Blue_ and PIPR_Normalized_ (Supplementary Table 1) between AD and controls.

To check the extent to which the control- and AD-regression lines of each pupillometric parameters with age deviate from parallel, the likelihood-ratio test was used (Supplementary Tables 2, 3 and Supplementary Figure 1). Likelihood-ratio test showed the existence of interaction age × group for melanopsin-mediated PIPR_Blue_ (Alzheimer’s group: β = -0.0042; 95% CI = -0.0073–0.0011; *r* = -0.5) and transient peak amplitude (Alzheimer’s group, blue flash: β = -0.0034; 95% CI = -0.0055–0.0014; *r* = -0.59; red flash: β = -0.0021; 95% CI = -0.0039–0.0003; *r* = -0.44) parameters with a significant correlation only in AD (Supplementary Tables 2, 3 and Supplementary Figures 1B,D,F).

Furthermore, Pearson’s correlation analysis was used to determine if there was a relationship between the calculated pupillometric parameters and OCT measures, MMSEc score, and disease duration (the latter two only for AD). There was no significant correlation between pupillometric parameters and OCT measurements, neither with MMSEc score and disease duration for AD patients. Mean comparisons of OCT measurements for all RNFL quadrants (temporal, superior, nasal, and inferior) and macular GCL + sectors (superotemporal, superior, superonasal, inferonasal, inferior, and inferotemporal) did not show significant differences between AD and controls (data not shown).

We also retrieved information regarding oral medications that could potentially interfere with pupillary responses, i.e., cholinesterase inhibitors for AD and beta-blockers for controls. Only a few controls (*n* = 4, 15.5%) were on beta-blockers (atenolol/metaprolol/bisoprolol), while 16 AD (61.5%) were on cholinesterase inhibitors drugs (donepezil/rivastigmine/galantamine). In controls, there were no differences in terms of pupillometric parameters between subjects taking beta-blockers and those not taking them (data not shown). No significant differences among AD subgroups (cholinergic-treated and cholinergic-untreated) for any PLR parameters were found (data not shown).

## Discussion

In this paper, we evaluated for the first time, using chromatic pupillometry, aimed at isolating the mRGC contribution (Park et al., 2017), the PLR in a cohort of 26 definite mild-moderate AD patients compared to a group of age- and gender-matched controls. In particular, to specifically target mRGC function, we evaluated the PIPR amplitude using intense (450 cd/m^2^) blue (472 nm) light stimuli. The PIPR, which is the most reliable marker of mRGC-mediated PLR, was not significantly different between AD and controls, but we found a significant difference in terms of transient PLR amplitude between AD and controls under dark-adaption. Concerning the PIPR, even though the difference between AD and controls was not significantly different, the AD group showed higher variability with five individuals having a PIPR_Blue_ amplitude value outside the 2 SD range from the control mean. Such variability in terms of disease severity has been already reported in terms of circadian measurements and optic nerve pathology (La Morgia et al., 2016) and might depend on the severity as well as on disease duration (Hatfield et al., 2004). In this study we included AD patients in a mild-moderate stage of the disease and disease severity and duration were on average lower than previously published cohorts (La Morgia et al., 2016). In rodents, six different mRGC subtypes were characterized, and PLR was mainly regulated by the Brn3b-positive M1 and non-M1 subtypes (Chen et al., 2011; Li and Schmidt, 2018). In humans, Hannibal and colleagues also identified six subtypes of mRGCs (M1, M2, M3, M4, giant M1, and giant displaced M1), unevenly distributed across the human retina and with distinct anatomical characteristics (Hannibal et al., 2017). We previously demonstrated in post-mortem AD retinas that mRGCs are lost in AD and amyloid pathology specifically affects these cells (La Morgia et al., 2016). However, it is not known whether in AD the neurodegenerative process affects a specific mRGC subtype, and in particular those mRGC contributing to the PLR.

Interestingly, we found a significant difference in terms of transient peak amplitude both under the rod-condition, using the short duration, narrowband pulse, and low intensity (0.001 cd/m^2^) blue light stimulus, and under the melanopsin-condition, but not in the cone-condition, overall pointing to a prominent rod-mediated response (McDougal and Gamlin, 2010; Kostic et al., 2016; Krishnan et al., 2020). Considering that mRGCs receive synaptic input from rods and cones through bipolar cells and a direct contact of rod bipolar cells via ribbon synapses in the ON layer of the IPL with mRGCs has been demonstrated in human retinas (Hannibal et al., 2017), this difference between AD and controls could suggest that in the early stages of the disease there is no obvious cell body dysfunction but possibly a dendropathy. This suggestion is based on the presence of mRGC dendrite pathology, previously reported in AD, with extensive morphological abnormalities in the spared mRGCs showing dendrite varicosities, patchy distribution of melanopsin, and reduced dendrite arborization (La Morgia et al., 2016). Dendritic degeneration has been also documented in RGCs of AD mouse model (Williams et al., 2013), and there are other disease models such as *OPA1*-related optic atrophy in which dendrites are the primary site of pathology (Williams et al., 2010). The different mRGC subtypes are distinctively connected to rods and cones and specifically modulated by various light conditions (Weng et al., 2013). Furthermore, the presence of a contact from amacrine cells and directly from rod bipolar cells via ribbon synapses on M1, M2, and M4 soma membrane and dendrites has been demonstrated in human retinas (Hannibal et al., 2017). The significantly reduced transient peak amplitude in conditions exploring the rod-contribution may thus suggest an altered contact between rods and mRGCs. It is possible to hypothesize that the rod response depends more on the distal dendrites, and consequently that the subsequent reduced dendritic arborization might interfere with the rod input out of proportion to the cones. However, we cannot exclude that the rod-mediated mRGC dysfunction in AD can be due to pathology specifically affecting rod-bipolar cells while possibly sparing cone-bipolar cells.

In summary, pathology often provokes compensation. This is particularly true with the central nervous system, which has many gain control circuits in place (Ostergaard et al., 2007; Do, 2019). Therefore, if disease, injury, or aging causes a reduction of units, there are many means for restoring the overall average mass effect. However, with coarser granularity, there is increased variability (Mendell, 2014). In this case, fewer mRGCs or even fewer dendritic circuits give less granularity in the system and a tendency for larger swings in the response. Thus, variability would precede decompensation into failure.

Baseline pupil size was not significantly different between AD and controls. It must be considered, however, that 16/26 AD patients were on acetylcholinesterase inhibitor drugs, and this might have an impact on the baseline pupil size. A few, small sample studies have reported the effect on the PLR of commonly used cholinergic AD drugs (Fotiou et al., 2000; Granholm et al., 2003). In one of these studies the authors did not find an effect of cholinergic medications on baseline pupil size, but demonstrated an increase in pupil constriction latency (Granholm et al., 2003). We also compared the PLR in AD patients taking and not taking these drugs and failed to demonstrate significant differences. Additional studies are needed to conclude a real effect of cholinergic medications on PLR and to clarify if different acetylcholinesterase inhibitors could have a different impact on PLR.

Previous studies investigated PLR in AD patients documenting reduced velocity, constriction amplitude, and increased latencies of PLR. These results were interpreted as related to the acetylcholine deficiency and parasympathetic dysfunction in AD (Prettyman et al., 1997; Fotiou et al., 2000, 2007, 2009; Tales et al., 2001; Granholm et al., 2003; Frost S. et al., 2013; Frost S.M. et al., 2013; Frost et al., 2017; Bittner et al., 2014; Chougule et al., 2019). Unfortunately, none of these studies were based on chromatic pupillometry protocol, and these results were not confirmed by more recent studies, which focused on early and pre-clinical stages of AD (Chougule et al., 2019). One chromatic pupillometry study evaluated pre-symptomatic AD cases (Oh et al., 2019). Oh and co-authors evaluated the PLR response using a similar pupillometric protocol in a cohort of 10 pre-symptomatic AD cases, defined on the basis of the cerebrospinal fluid markers, and they did not demonstrate a significant difference between pre-symptomatic AD cases and controls (Oh et al., 2019). However, congruent to the current findings, higher variability of PLR was documented in the AD group (Oh et al., 2019). Moreover, in this study the authors used only the 2.3 log cd/m^2^ photopically-matched red and blue stimuli (Oh et al., 2019). Similarly, Van Stavern and co-authors, using a white light stimulus, did not show any difference between preclinical AD subjects (defined by CSF markers) and normal aging controls in any of the PLR parameters examined (Van Stavern et al., 2019). Very recently, Kawasaki et al. (2020), using a different chromatic pupillometry protocol under photopic conditions, failed to demonstrate, similarly to our results, a significant difference between early AD and controls in terms of PIPR response.

We also demonstrated a significant correlation of the PIPR amplitude and transient peak amplitude (melanopsin-condition, 450 cd/m^2^) with age only in the AD group, which is in line with previous results pointing to an accelerated aging process in AD (La Morgia et al., 2016). Data on PLR in relation to age are not conclusive even though the majority of papers failed to reveal a significant difference of PLR in relation to the aging process. However, the controls included in these studies were younger than 70, and this might explain the absence of significant impairment of the pupil response (Adhikari et al., 2015a; Rukmini et al., 2017). Our results are in line with the observation of mRGC loss with age (Semo et al., 2003; La Morgia et al., 2016; Esquiva et al., 2017).

We did not find a correlation between pupil metrics and OCT parameters nor with disease severity or duration. Moreover, we did not find any significant difference in terms of RNFL and GCL + thickness between AD and controls. Any effect specific to mRGC loss would have been swamped by regular RGCs in these measures. Further, these results can be explained by the inclusion of milder cases with shorter disease duration. A recent SS-OCT study evaluating a large cohort of AD cases failed to demonstrate a significant difference in terms of RNFL between AD and controls (Sanchez et al., 2018).

Overall, the current chromatic pupillometry findings in a cohort of mild-moderate AD patients did not demonstrate a clear mRGC-driven pupil dysfunction but are rather consistent with a dendropathy in the early stage of the disease, supported by our previous post-mortem studies of AD retinas. Early pathology, while still in the range of compensatory mechanisms, often manifests as variability. Further studies including more severe and with longer disease duration AD cases are needed to further explore this hypothesis. Such studies may also clarify whether the PLR can be used as a tool evaluating the progression of the disease and eventually the efficacy of therapies in AD.

## Data Availability Statement

The raw data supporting the conclusion of this article will be made available from the corresponding author, without undue reservation, to any qualified researcher.

## Ethics Statement

The studies involving human participants were reviewed and approved by the EC Interaziendale Bologna-Imola #16032. The patients/participants provided their written informed consent to participate in this study.

## Author Contributions

MR: acquisition, analysis and interpretation of data, and drafting and revising the work. MS, MD, SC, MC, GA, and CL: data acquisition and revising the work. MR and CZ: statistical data analysis. MR, MC, GC, RL, AS, VC, JP, and CL: contributed to interpretation of the data. MR, GC, VC, JP, and CL: design of the work. MS, MD, SC, MC, GA, GC, CZ, RL, AS, VC, JP, and CL: revising the work and provided approval for publication of the content. All authors contributed to the article and approved the submitted version.

## Conflict of Interest

The authors declare that the research was conducted in the absence of any commercial or financial relationships that could be construed as a potential conflict of interest.
