# Supplementary material for: Chromatic Pupillometry Findings in Alzheimer’s Disease
Source: Front Neurosci. 2020 Aug 11;14:780. doi: 10.3389/fnins.2020.00780 (PMC7431959; doi:10.3389/fnins.2020.00780)
Supplement: Supplementary file 1 [file Data_Sheet_1.docx]

## Supplementary Tables

**Supplementary Table 1. Mean (±standard error of the mean) age-adjusted pupillometric parameters in controls and AD (ANCOVA).**

|  | Control | AD | p-value |
| --- | --- | --- | --- |
| ROD  N | 24 | 26 |  |
| Baseline normalized pupil size (0.001 cd/m^2^, 472 nm)  Mean±SE | 5.7±0.24 | 5.8±0.23 | 0.81 |
| Transient Peak Amplitude (0.001 cd/m^2^, 472 nm)  Mean±SE | 0.27±0.01 | 0.22±0.01 | 0.006* |
| MPS  N | 24 | 26 |  |
| Baseline normalized pupil size (450 cd/m^2^, 472 nm)  Mean±SE | 5.5±0.2 | 5.6±0.2 | 0.67 |
| Transient Peak Amplitude _Blue_ (450 cd/m^2^, 472 nm)  Mean±SE | 0.54±0.01 | 0.51±0.01 | 0.02* |
| Transient Peak Amplitude _Red_ (450 cd/m^2^, 632 nm)  Mean±SE | 0.49±0.01 | 0.45±0.01 | 0.006* |
| PIPR _Blue_ (450 cd/m^2^, 472 nm)  Mean±SE | 0.49±0.02 | 0.46±0.02 | 0.17 |
| PIPR _Normalized_ (PIPR _Blue_, 472 nm – PIPR _Red_, 632 nm)  Mean±SE | 0.36±0.01 | 0.36±0.01 | 0.89 |
| CONE  N | 22 | 20 |  |
| Baseline normalized pupil size (10 cd/m^2^, 632 nm)  Mean±SE | 3.2±0.1 | 3.3±0.1 | 0.83 |
| Transient Peak Amplitude (10 cd/m^2^, 632 nm)  Mean±SE | 0.23±0.01 | 0.22±0.01 | 0.61 |

Values are given as n (absolute frequency) or mean ± standard error. AD = Alzheimer’s Disease; PIPR = Post-Illumination Pupil Response. p-values are shown for the Analysis of covariance (ANCOVA). Significant p-values are shown with the asterisk (*).

**Supplementary Table 2. Likelihood-ratio test comparing the goodness of fit of two competing ANCOVA models (with and without interaction term Group×Age) of each pupillometric parameters.**

|  | p-value |
| --- | --- |
| ROD |  |
| Baseline normalized pupil size (0.001 cd/m^2^, 472 nm) | 0.349 |
| Transient Peak Amplitude  (0.001 cd/m^2^, 472 nm) | 0.708 |
| MPS |  |
| Baseline normalized pupil size  (450 cd/m^2^, 472 nm) | 0.374 |
| Transient Peak Amplitude _Blue_  (450 cd/m^2^, 472 nm) | 0.098* |
| Transient Peak Amplitude _Red_  (450 cd/m^2^, 632 nm) | 0.132* |
| PIPR _Blue_  (450 cd/m^2^, 472 nm) | 0.072* |
| CONE |  |
| Baseline normalized pupil size  (10 cd/m^2^, 632 nm) | 0.624 |
| Transient Peak Amplitude  (10 cd/m^2^, 632 nm) | 0.185 |

*Likelihood-ratio test < 0.15

**Supplementary Table 3. Linear regression coefficients (β) with 95% Confidence Interval (95% CI) between pupillometric parameters and age (years) for the two groups (controls and AD).**

| MPS | β control  (95% CI) | β AD  (95% CI) |
| --- | --- | --- |
| Transient Peak Amplitude _Blue_  (450 cd/m^2^, 472 nm) | -0.0009  (-0.0028 / 0.001) | -0.0034  (-0.0055 / -0.0014) |
| Transient Peak Amplitude _Red_  (450 cd/m^2^, 632 nm) | -0.0001  (-0.0019 / 0.0018) | -0.0021  (-0.0039 / -0.0003) |
| PIPR _Blue_  (450 cd/m^2^, 472 nm) | 0.0004  (-0.0035 / 0.0043) | -0.0042 (-0.0073 / -0.0011) |

## Supplementary Figures


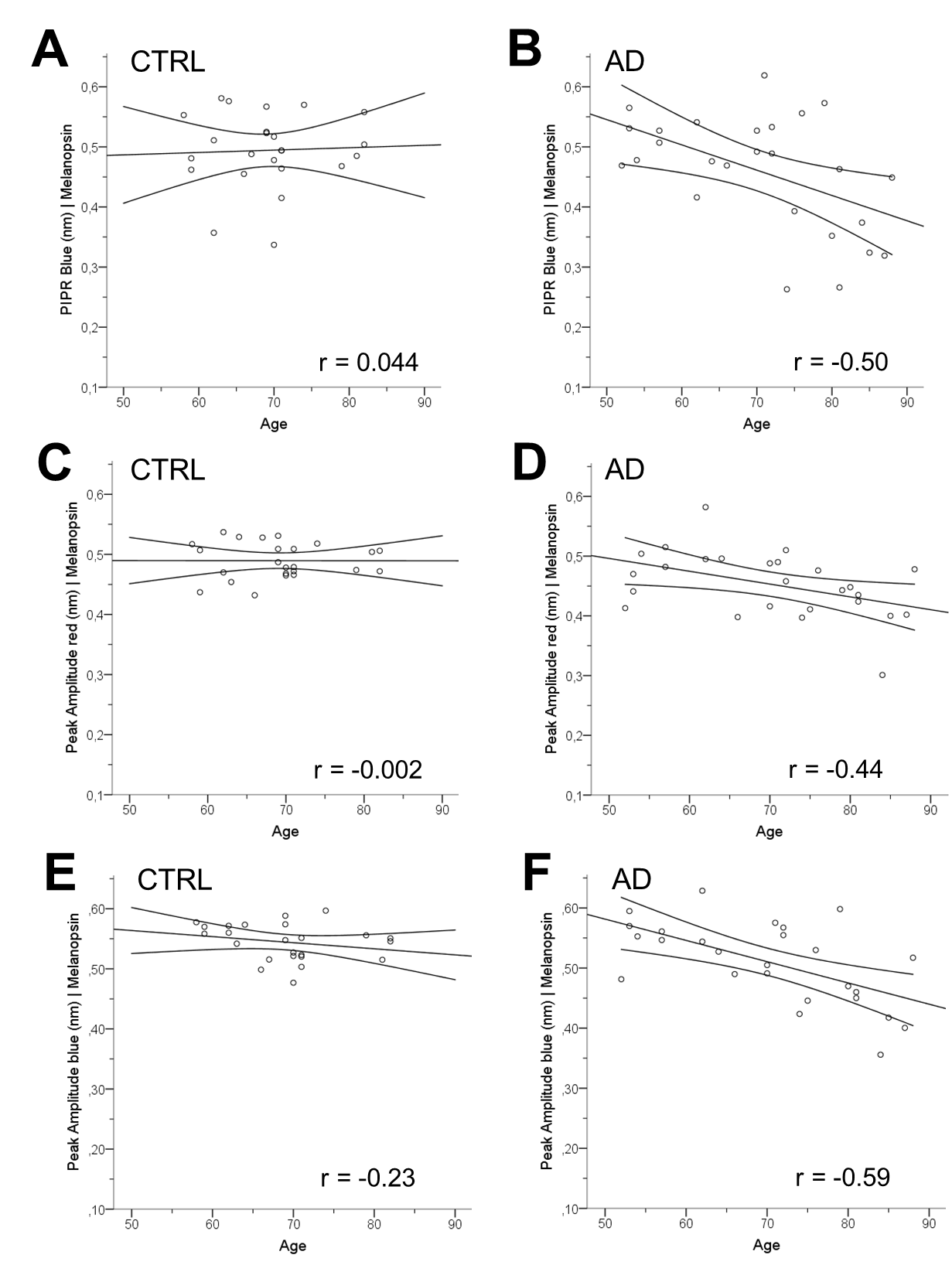


**Supplementary Figure 1. Correlations of pupillometric parameters with age for melanopsin-condition.** Panels **A** and **B** show scatterplots displaying the melanopsin-mediated PIPR amplitude (450 cd/m^2^, 472 nm-blue; y-axis) against age (x-axis) with the regression line of best fitting and individual confidence intervals in control (A) and AD (B). Panels **C** and **D** show scatterplots displaying correlation of the transient peak amplitude (450 cd/m^2^, 632 nm-red; y-axis) with age (x-axis) with the regression line of best fitting and individual confidence intervals in controls (C) and AD (D). Panels **E** and **F** show scatterplots displaying correlation of the transient peak amplitude (450 cd/m^2^, 472 nm-blue; y-axis) with age (x-axis) with the regression line of best fitting and individual confidence intervals in controls (E) and AD (F).
